# Fabrication of a Novel Protein Sponge with Dual-Scale Porosity and Mixed Wettability Using a Clean and Versatile Microwave-Based Process

**DOI:** 10.3390/ma14092298

**Published:** 2021-04-29

**Authors:** Judith Wemmer, Loredana Malafronte, Socrates Foschini, Aline Schneider, Christian M. Schlepütz, Martin E. Leser, Martin Michel, Adam Burbigde, Erich J. Windhab

**Affiliations:** 1 Laboratory of Food Process Engineering, Institute of Food, Nutrition and Health, ETH Zurich, Schmelzbergstrasse 9, 8092 Zurich, Switzerland; judith.wemmer@hest.ethz.ch (J.W.); socrates.foschini@hest.ethz.ch (S.F.); aline.schneider@hest.ethz.ch (A.S.); 2 Swiss Light Source, Paul Scherrer Institute, 5232 Villigen, Switzerland; christian.schlepuetz@psi.ch; 3 Société des Produits Nestlé S.A.,Nestlé Research, Route du Jorat 57, 1000 Lausanne, Switzerland; martin.leser@rdls.nestle.com (M.E.L.); martin.michel@rdls.nestle.com (M.M.); adam.burbidge@rdls.nestle.com (A.B.)

**Keywords:** sponge, protein, microwave, scaffolding, delivery, food, pharma, drying, absorption, porosity

## Abstract

An open-porous protein sponge with mixed wettability is presented made entirely from whey proteins and with promising applications in biomedicine, pharmaceutical, and food industry. The fabrication relies on an additive-free, clean and scalable process consisting of foaming followed by controlled microwave-convection drying. Volumetric heating throughout the matrix induced by microwaves causes fast expansion and elongation of the foam bubbles, retards crust formation and promotes early protein denaturation. These effects counteract collapse and shrinkage typically encountered in convection drying of foams. The interplay of high protein content, tailored gas incorporation and controlled drying result in a dried structure with dual-scale porosity composed of open macroscopic elongated foam bubbles and microscopic pores in the surrounding solid lamellae induced by water evaporation. Due to the insolubility and mixed wettability of the denatured protein network, polar and non-polar liquids are rapidly absorbed into the interconnected capillary system of the sponge without disintegrating. While non-watery liquids penetrate the pores by capillary suction, water diffuses also into the stiff protein matrix, inducing swelling and softening. Consequently, the water-filled soft sponge can be emptied by compression and re-absorbs any wetting liquid into the free capillary space.

## 1. Introduction

Porous solids, aerogel-like or sponge-like structures are being applied in cosmetics, electrotechnics, food, chemical, biomedical and pharmaceutical applications [1,2], for various technical challenges, such as for water treatment [3], tissue engineering [4,5], drug delivery [6] or catalysis [7]. The high porosity of such structures leads to a high surface-to-volume ratio, to a low density, and thus light weight. When open pores are produced, it may result in the ability to passively or actively absorb and release liquids, such as in a sponge, given the structure does not disintegrate upon contact with liquids. Furthermore, the application of biopolymers, such as polysaccharides and proteins, as bulk material favors the biodegradability, renewability and edibility of related materials [2]. The interest in polysaccharide and protein-based aerogels or sponge-like materials is growing, mainly for drug delivery, and lately as advanced material for functional food, i.e., as hosting matrix for active compounds and nutraceuticals. Since the 1990s [8], polysaccharide-based aerogels have been systematically investigated and unique properties have been achieved starting from low cost raw materials, such as agar, starch, cellulose, pectin, and alginate [9,10]. For instance, cellulose aerogels are the most common and they have been extensively proven to be suitable as stimuli-responsive materials thanks to their ability to alter their chemical and physical properties upon triggering, such as variations in pH, temperature and ionic strengths; thus, they are relevant as drug carriers, for the development of scaffolds and other biomedical applications [11]. Alginate and starch aerogels were applied for the development of active food packaging when loaded with an antioxidant and antimicrobial agent [12], and as support for poorly water-soluble vitamins so as to increase their bioavailability [13,14]. Contrarily, studies on protein-based aerogels or sponge-like materials are still limited. Silk fibroin aerogels showed potential as scaffolds for tissue engineering [15]. Denatured soy proteins were tested to obtain aerogels with and without the addition of nanocellulose. They did not dissolve in water and they were able to absorb polar and non-polar solvents [16]. Similarly, aerogels based on whey proteins were found to be water insoluble and applicable as drug carriers. Their microstructure and mechanical properties depend on the pH during gelation and their drying process [17]. Whey protein aerogels have also been proven to be effective carriers of sensitive oils, i.e., fish oil, together with sodium caseinate and egg white protein-based aerogels [18].

The established approaches for the fabrication of biopolymer-based sponge-like structures require freeze-drying [17,19,20,21,22], solvent exchange and supercritical carbon dioxide drying [17,23,24], or solvent exchange and vacuum drying [25], partly in combination with covalent cross-linking through chemical additives [19,21,22,25] or heating [17,23]. These gentle drying methods allow to minimize shrinkage and yield highly porous materials. However, they tend to be time-, energy-, and cost-intensive and hence not attractive for large scale production [26,27]. A more scalable processing route was proposed by Perez-Puyana et al. [28]. They developed a process based on injection molding for protein-based biodegradable absorbents, but it required the addition of ≥50% fillers and plasticizers. 

Here, we demonstrate a simple and upscalable fabrication process for open-porous sponge structures, purely based on proteins, without the need for additives. A concentrated whey protein isolate (WPI) dispersion was foamed and dried using superimposed microwave and convection heating, leading to a protein sponge with a dual-scale porosity and a mixed wettability. Effects of operating conditions on macro- and microstructure of the final sponge were studied. Microstructure was investigated using scanning electron microscopy (SEM) and synchrotron computed microtomography (μ-CT). The mixed wettability signifies the ability to passively absorb polar and non-polar liquids, as defined in the frame of complex porous materials, such as rocks [29,30], and here demonstrated in absorption tests. The absorption tests comprised evaluation of the absorption capacity and kinetics. In addition, the mechanical properties of the final sponges were studied before and after polar and non-polar liquid absorption.

## 2. Materials and Methods

### 2.1. Materials

Whey protein isolate (Rapid Whey Protein Isolate 894) was provided by Fonterra Co-operative Group Limited (Auckland, New Zealand).

### 2.2. Foam Preparation and Drying

An amount of 40 wt% of WPI powder was manually dispersed in tap water and stored overnight at approximately 5 °C for hydration prior to foaming in a dynamic membrane gas-dispersing system (FOAMEM, Kinematica AG, Malters, Switzerland). The foaming head was composed of two concentric cylinders with a gap width of 3 mm. The inner full metal cylinder was rotated, while the outer static cylinder consisted of a porous sinter-metal membrane with a pore diameter between 2 and 5 μm. The protein–water mix was axially pumped through the gap at a flow rate of 40 mL min^−1^. Pressurized nitrogen gas at 2.5 bar and at a flow rate of 70 mL min^−1^ was pressed through the porous sinter membrane. Bubbles were detached into the continuous phase at a rotation speed of 6000 rpm. The double jacket surrounding the foaming head was cooled to 10 °C. A gas volume fraction of 70 vol% was incorporated into the WPI–water mixture with a bubble size of d_50,0_ = 54 μm and SPAN = ((d_90,0_ − d_10,0_)/d_50,0_) of 1.28. A volume of 24 mL of foam filled cylindrical transparent polypropylene molds of 27.5 mm in diameter and 86 mm in height. Four samples were placed onto a Teflon plate and dried by a combination of microwave and convection heating at 60 °C/50 W for 3 h or 60 °C/100 W for 2 h, or by pure convection at 100 °C for 3 h in a combi-steam MSLQ oven (V-Zug AG, Zug, Switzerland). The long drying times were chosen to ensure complete water removal as kinetically restricted by the cylindrical mold. 

### 2.3. In-Line Temperature Measurement

The sample temperature was measured during the drying process with a fiber-optic temperature sensor (TS3 and FOTEMP4, Optocon AG, Dresden, Germany) with an accuracy of ±0.2 °C. The sensors were inserted through the bottom of the cylindrical molds to a height of 32 mm. In the radial position, the sensors were placed in the center and at half radius position to measure related local temperatures. 

### 2.4. In-Line Expansion Determination

Expansion was observed by taking pictures with a camera (Lumix DMCFZ1000, Panasonic, Osaka, Japan) placed approximately 10 cm in front of the glass door of the oven. The camera and oven glass door were shielded from surrounding light sources with black cloth. The oven light was turned on. The sample height was measured in the mold center as pixel number related to the known mold height.

### 2.5. Scanning Electron Microscopy

Specimens were cut with razor blades into slices approximately 2 mm in height and glued to the sample holder with double-sided tape. The specimen surface was coated with platinum-palladium (8 nm) with a sputter coater (CCU-010 Metal, Safematic, Zizers, Switzerland). Images were taken with a field-emission scanning electron microscope (SU5000, Hitachi, Tokyo, Japan) at an accelerating voltage of 3 kV.

### 2.6. Absorption Test

The absorption capacity was determined by placing cylindrical pieces of the dry protein sponge into petri dishes with MilliQ water or silicon oil (M3, Carl Roth GmbH + Co. KG, Karlsruhe, Germany), waiting until the liquid had reached the upper edge of the sample and weighing. The absorption kinetics were assessed by measuring the time from the first contact with the liquid until the liquid had reached the upper edge of the sample. For sequential absorption of water and oil, a sponge piece was cut into half and soaked in either water or silicon oil overnight to ensure complete absorption and swelling. The filled sponges were placed in petri dishes with either water colored with blue colorant (E133) or silicon oil stained with wax red. To avoid evaporation, the water-filled sponge was placed in a desiccator above an open beaker with water.

### 2.7. Synchrotron Computed Microtomography (μ-CT)

Synchrotron X-ray tomographic microscopy was performed at the TOMCAT beam-line X02DA of the Swiss Light Source, Paul Scherrer Institute (Villigen, Switzerland). The dried sponge samples were cut into cylinders of 5 mm in diameter using a plastic straw, attached to a sample holder with wax and investigated in a dry state, as well as after addition of decane or water. Decane was mixed with 10 wt% 1,10-diiododecane (Sigma Aldrich, St. Louis, MO, USA) for enhanced contrast. A total of 1500 projections were acquired over 180° at an X-ray energy of 12 keV and an exposure time of 250 or 120 ms with a pco.Edge 5.5 camera (pco, Kelheim, Germany). Utilization of a 10x optical magnification of the X-ray image produced on the 20 μm thick LuAG:Ce scintillator resulted in an effective pixel size of 0.65 µm and a view field of 1.4 × 1.7 mm^2^. Reconstruction of the tomographic volume data sets was performed with gridrec [31] using a propagation-based phase contrast algorithm [32] to enhance the contrast between the solid material, air and liquid phase. Binarization and contrast enhancement were performed using Avizo (ThermoFischer Scientific, Waltham, MA, USA) and ImageJ [33].

### 2.8. Mechanical Analysis

Sponge cylinders of 20 mm in height were cut using a sharp blade, 20 mm above the bottom of the dried sponge samples. The cylindrical pieces, either in a dried state or with absorption of excess MilliQ water or silicon oil, were uniaxially compressed in the expansion direction by 70% at a velocity of 0.02 mm s^−1^ with a texture analyzer (TA.XT Plus, Stable Micro Systems, Godalming, UK) equipped with a 50 N (for water-filled) or 500 N (for oil-filled and dried) load cell and a cylindrical plate of 100 mm in diameter.

### 2.9. Statistical Analysis

A two-sample *t*-test with unequal variances was performed to compare the density of the protein sponges at the different operating conditions. The test was also performed to assess the differences between oil and water absorption capacity and filled pore fraction. The significant level was set to 0.05.

## 3. Results and Discussion

### 3.1. Fabrication Process of the Protein Sponge

**Figure 1:**
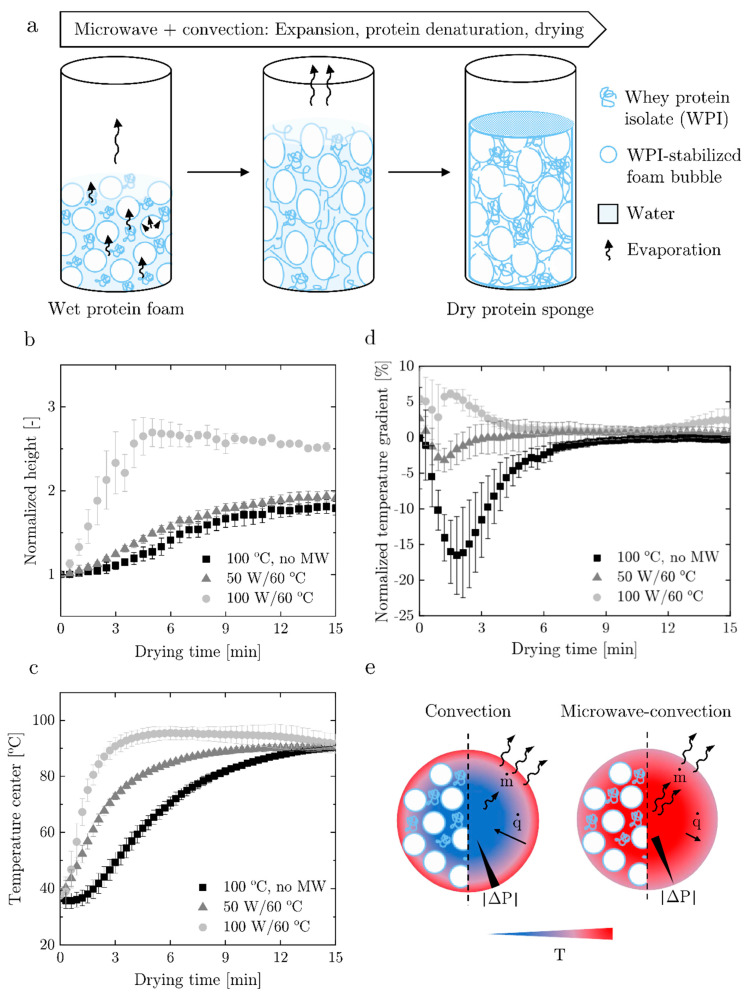
Microwave-convection drying of protein foam: (**a**) schematic illustration of the drying process to turn a wet protein foam based on whey protein isolate into a dry protein sponge; involving expansion, protein denaturation and drying induced by superimposed microwave and convection heating in an open top cylindrical mold. (**b**) Expansion kinetics at different drying conditions; (**c**) center temperature at different drying conditions; (**d**) normalized temperature gradient within the foam in radial direction at different drying conditions; and (**e**) schematic illustration of temperature distribution T, heat flux (q), water flux (m) and absolute pressure differences |ΔP| over the radial cross-section of the foam sample at the beginning of the drying process with and without superposition of microwaves.

A highly concentrated whey protein isolate (WPI) dispersion, composed of 40 wt% WPI in water, was foamed with a dynamically enhanced membrane foaming apparatus [34] to adjust the gas volume fraction to 70 vol%. The wet foam was molded and dried in an oven using controlled superimposed microwave (MW) and convection heating to yield a dry, porous protein sponge. Three operating conditions were investigated: (i) hot air at 100 °C (100 °C, no MW), (ii) hot air at 60 °C and microwave heating at 50 W (50 W/60 °C), and (iii) hot air at 60 °C and microwave heating at 100 W (100 W/60 °C). Heating of the foam into the cylindrical molds led to drying, protein denaturation (i.e., protein unfolding and aggregation) and to foam expansion, due to the expansion of the gas phase and, more importantly, to steam generation, as illustrated in Figure 1a. The expansion in the partially confined space of the mold promotes elongation of the product and its foam bubbles. 

The expansion kinetics, the temperature profiles at the center of the sample and the temperature gradient in the sample as a function of the drying time are reported in Figure 1b–d. A comparison of expansion kinetics under different drying conditions (Figure 1b) revealed a faster and higher expansion with increasing power of the superimposed microwave radiation compared to pure convective drying (100 °C, no MW). The increase in the height of the final sample amounted to about 1.7 times in the case of pure convection, almost2 times for 50 W/60 °C and 2.5 times for 100 W/60 °C. Monitoring of the sample temperature during drying showed that the center temperature increased faster when microwave heating was applied at an increased microwave power (Figure 1c). In addition, the temperature difference between the center (T_c_) and edge (T_e_) in the radial direction, plotted as a normalized temperature gradient, (T_c_ − T_e_)/T_c_ (Figure 1d), showed that pure convective drying resulted in an initially higher edge temperature with a relative gradient of up to −17%, which equilibrated as the heat was conducted to the center. Instead, superposition of microwave heating led to a minor temperature gradient throughout the sample of up to −3% at 50 W and a maximum of +6% at 100 W. The higher edge temperature, as in case of pure convection, indicated that the surface of the sample dried faster than the center, causing a crust or skin formation, and high capillary pressure differences in the surface layer, consequently limiting expansion in the initial stage of drying [35]. In contrast, microwave radiation induced motion of the water dipoles, which resulted in dissipation of energy as heat. This led to volumetric heating, immediate internal steam generation and expansion [36], demonstrated by the smaller temperature gradient from the edge to the center of the sample when compare to pure convection (Figure 1d) and significant longitudinal expansion (Figure 1b). The drying mechanism is schematically shown in Figure 1e, convective heating primarily promoted transport of water ($\dot{m}$) from the surface to the surrounding while internal water transport was limited by the conductive heat transfer ($\dot{q}$). Gentle volumetric heating with microwaves at low power leads to a moderate internal pressure increase, promoting transport of water and vapor from the center to the sample surface, preventing crust formation and counteracting evaporation-induced shrinkage [37]. The immediate volumetric temperature increased at a high water content promoted protein denaturation throughout the foam structure and thus caused solidification and stabilization of the interfacial protein layer, and the formation of an aggregated protein network in the continuous phase (lamella). The related increase in mechanical stability prevented pore collapse. The delayed crust formation may have also prolonged expansion, which promoted coalescence and the break-up of bubbles, generating a highly open-porous structure.

### 3.2. Structure of the Dried Sponge

**Figure 2:**
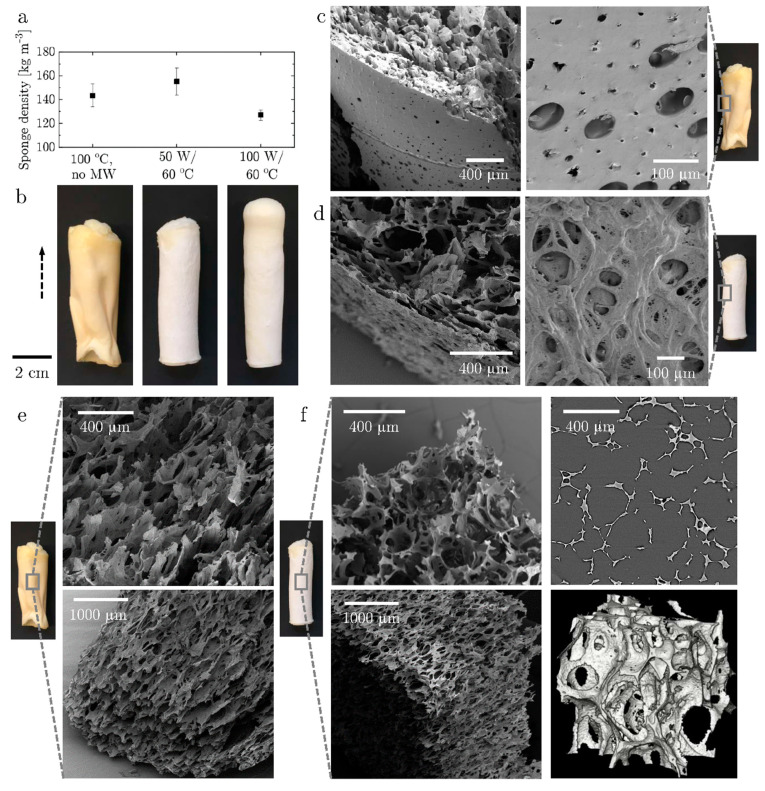
Macro- and microstructure of the protein sponge dried with and without superposition of microwave heating: (**a**) average densities of dried protein sponges at different drying conditions; (**b**) macroscopic images of dried protein sponges at different drying conditions (the dashed arrow indicates the expansion direction); (**c**) surface layer formed after drying at 100 °C without microwave heating; (**d**) surface layer of protein sponge dried at 50 W/60 °C; (**e**) inner structure formed after drying at 100 °C without microwave heating; (**f**) inner structure of protein sponge dried at 50 W/60 °C investigated by SEM (left) and μ-CT (right). The corresponding SEM images of the protein sponge dried at 100 W/60 °C are found in the Supporting Information Figure A (Supplementary Materials).

The structure of the sponges at the end of the drying process has been investigated at a micro- and macro-scale and in relation to their average density (Figure 2).

Moderate longitudinal expansion and radial shrinkage at the end of the drying process led to similar sample densities in the range of 125–165 kg·m^−3^ at all operating conditions (Figure 2a). This is equivalent to porosities of 88–91% at a solid density of 1400 kg·m^−3^ [38]. According to the two-sample t-test assuming unequal variances, the samples dried at 100 W/60 °C have significantly lower density than the samples dried at 50 W/60 °C (*p*-value = 0.003). However, the microwave-dried samples do not significantly differ in density compared to the convection-dried samples. Aerogels with bulk density in the range of 112–400 kg·m^−3^ have been obtained by freeze drying and supercritical drying whey protein hydrogels [17,39]. Hence the density of the porous material presented in this study is comparable.

**Figure 3:**
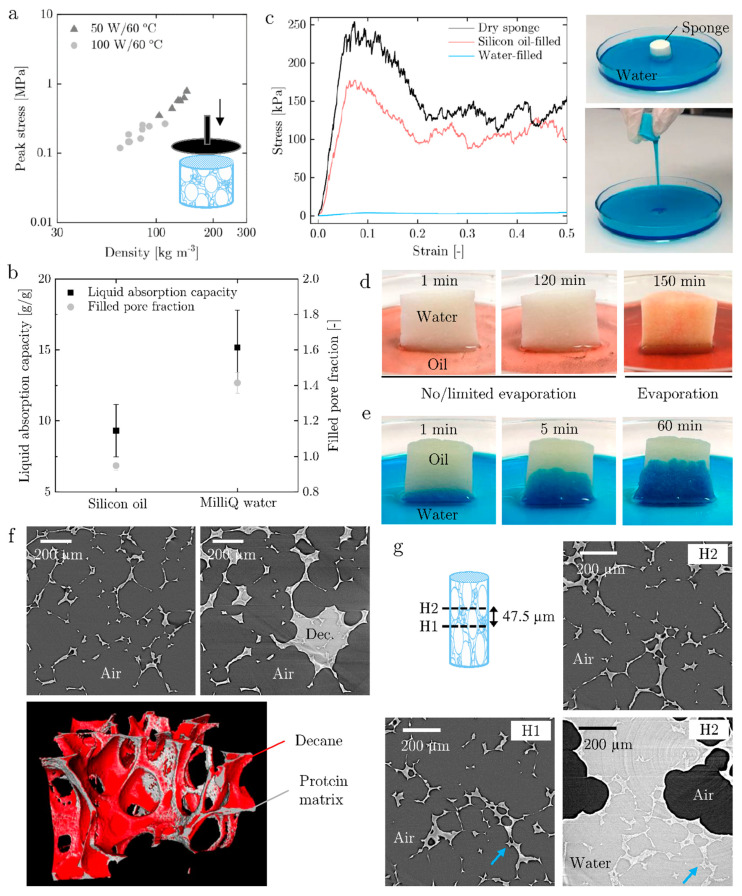
Mechanical properties and liquid absorption of microwave-convection dried protein sponges: (**a**) peak stress in uniaxial compression as function of sponge density; (**b**) liquid absorption capacity and fraction of filled pore volume with silicon oil and MilliQ water for sponges dried at 100 W/60 °C; (**c**) stress response of a sponge dried at 100 W/60 °C in dry state, filled with low-viscous silicon oil, and filled with water in uniaxial compression (left), and images of water absorption and water release by compression (right); (**d**) silicon oil absorption into water-filled 100 W/60 °C sponge with and without limitation of water evaporation (oil stained in red); (**e**) water diffusion and absorption into silicon oil-filled 100 W/60 °C sponge over time (water stained with blue food colorant); (**f**) μ-CT images of 100 W/60 °C sponge in dry state and wetted by decane at the same position (top) and a volume-representation of the binarized μ-CT data, superimposing the volume occupied by decane colored in red with the protein backbone colored in grey (bottom); (**g**) μ-CT images of 100 W/60 °C sponge in dry state at sample height **H2** (top right) and sample height **H1** (bottom left) and after absorption of water at sample height **H2** (bottom right).

While all applied drying conditions led to similar densities, macroscopic and microscopic structures differed significantly with and without superposition of microwave radiation. As depicted in Figure 2b, convection-dried samples showed large wrinkles on the surface caused by high pressure differences during heterogeneous heating. At 50 W/60 °C, the shape resembled the utilized mold with a minor shrinkage in diameter. At 100 W/60 °C, expansion in height and radial shrinkage were more pronounced. The differences in microstructure between convection-dried and microwave-convection dried samples are depicted in SEM images. The SEM images of the samples 100 °C/no MW and 50 W/60 °C are shown in Figure 2c–f, and the corresponding images of the 100 W/60 °C samples are found in the Supporting Information, Figure A. The dominating water transport at the surface in pure convection drying caused the formation of a dense and thick crust, which is perforated to allow steam release (Figure 2c). In contrast, the continuous water transport from the center to the surface with superimposed microwave led to the formation of a thin, meshed skin with microscopic and macroscopic pores (Figure 2d). The inner structure in the convection-dried sample showed parallel solid protein sheets rather than spherical pores (Figure 2e). This might be due to an enhanced longitudinal evaporation of water. The slow conduction of heat to the core could not immediately fix the foam structure due to temperatures that were too low for protein denaturation, leaving more time for foam destabilization, and the promotion of the formation of a crust that hindered radial evaporation of water. With superposition of microwaves, the immediate and even temperature increased at 50 W/60 °C and 100 W/60 °C caused homogeneous fixation of the foam structure through protein denaturation, providing high enough mechanical stability to withstand evaporation-induced capillary stresses (Figure 2f, Supporting Information Figure A). The foam bubble diameter however increased from a mean of approximately 50 μm after foaming to up to 350 μm after drying and the bubble shape elongated through expansion in the partially confined space of the cylindrical mold (Figure 2f, bottom left, Supporting Information Figure A). Bubble expansion and contraction of the continuous phase caused bubble coalescence and break-up of the interfacial layers. Moreover, the μ-CT axial slice image and the volume representation of the tomographic data of the samples produced at 50 W/60 °C (Figure 2f, right) and at 100 W/60 °C (Figure 3f) revealed smaller cavities in the lamellar space between the macroscopic foam bubbles. The meshed and microporous structure of the solid protein matrix is most likely induced by local steam generation throughout the continuous phase.

In this work, we assume that the combination of foaming and drying of the protein network leads to the formation of sponges with a dual-scale porosity, consisting of macroscopic pores due to foaming (Figure 2) and micro-porous lamellae due to water evaporation (drying). The superimposition of the microwave heating promotes the production of a homogeneous and highly interconnected porous structure. Whilst pure convection leads to a heterogeneous and poorly connected porous material. 

### 3.3. Mechanical Properties and Absorption Performance of the Dried Sponge

The dried protein sponges were characterized in terms of their mechanical properties and absorption performance (Figure 3). The mechanical properties were investigated performing compression tests. The absorption performance or sponge-like behavior was studied in terms of absorption capacity and kinetics using polar (i.e., water) and non-polar (i.e., silicon oil) liquids. 

Figure 3a shows the peak stress in compression as a function of the density for samples produced at 50 W/60 °C and 100 W/60 °C. Mechanical stability increases with higher density, which corresponds to a low microwave power, meaning that the mechanical properties of the final product can be tailored by adjustment of drying parameters. The analysis of the mechanical properties of the sponge dried by pure convection could not be performed due to the heterogeneous structure and thick crust. 

The results of the absorption tests are reported for the 100 W/60 °C samples, the 50 W/60 °C behaved similarly. Absorption tests demonstrate that the microwave-convection-dried structure instantly absorbs water, oil and other solvents (decane, ethanol, toluene etc.) without disintegrating comparable to a sponge (Figure 3b–d and Supporting Information Videos S1–S3). This can be attributed to the insolubility of the denatured, irreversibly cross-linked protein network [17,40] and the highly interconnected capillary system. The less interconnected convection-dried sample breaks into chunks upon reconstitution and the thick crust and heterogeneous pores do not permit instant liquid absorption. The ability to absorb both polar and non-polar liquids, referred to as mixed wettability, is provided by the amphiphilic character of the proteins and the high and open porosity. Moreover, the microwave-convection-dried protein sponges did not disintegrate when stored for at least a month in water, oil or ethanol. 

The absorption capacity is higher for MilliQ water (15 g/g) compared to silicon oil (9 g/g) (Figure 3b, left, y-axis), and their difference is significant (*p*-value = 0.15). Penetration of water into the structure leads to an increase in total volume, referred to as swelling. While the oil fills up 0.95 of the pore volumes, water fills up 1.4 times the pore volume (Figure 3b, right, y-axis), and the results are significantly different (*p*-value = 0.0001). Additionally, water absorption causes softening of the solid structure due to its plasticizing effect. As shown by uniaxial compression in Figure 3c, the initially elastic-brittle porous solid becomes soft and plastically deforming when filled with water but stays mechanically stable and brittle when instead filled with silicon oil. While the oil-filled material fractures upon compression, the water-filled sponge can be emptied by compression and refilled reversibly, reaching an only slightly lower volume and liquid absorption capacity after multiple cycling (Figure 3c, right). 

Absorption kinetics experiments showed a similar liquid penetration velocity d*l*/d*t* for water and low-viscous silicon oil of (d*l*/d*t*)_water_ = 2.2 mm·s^−1^ and (d*l*/d*t*)_oil_ = 1.5 mm·s^−1^ into sponge cylinders of 20 mm height. The Washburn equation [41] (Equation (1)) for liquid penetration into a capillary of radius r and length l, with given liquid viscosities η, η_water_ = 0.001 Pa·s and η_silicon oil_ = 0.003 Pa·s, and surface tensions γ, γ_water_ = 0.073 N·m^−1^ and γ_silicon oil_ = 0.019 N·m^−1^, predicts any of the following effects: (i) a smaller radius of water-absorbing pores at the same wetting angle θ, (ii) a higher wetting angle, equivalent to lower wettability, for water at same pore radius, (iii) same initial pore radius, which decreases over time during water absorption due to swelling of the lamellae [42], or (iv) a combination of several of these effects.

(1) $$\frac{dl}{dt}=\frac{r\cdot \gamma \cdot cos\theta }{4\cdot \eta \cdot l}$$

Subsequent absorption of water and silicon oil (Figure 3d,e) suggests both water and oil penetrate into the same open capillary system. When completely filled with water (Figure 3d), silicon oil is only absorbed if the water leaves the pores through evaporation such as in unsaturated environment. In contrast, water slowly penetrates the oil-filled sponge by diffusing into the solid matrix supposedly on a molecular level (Figure 3e), leading to softening and potentially expansion. This results in an increase in pore volume visible as macroscopic swelling, and hence to absorption of water into the newly available pore space and partial leaching of oil possibly to reduce interfacial area between both phases. The μ-CT images in Figure 3f showing the dry sponge (top left) and the identical sample partly filled with decane (top right) confirm that the solid structure is unaffected by absorption of the non-watery liquid. Binarization and superimposing of the μ-CT images with and without decane (Figure 3f, bottom) elucidate that the low amount of added decane spreads as a film over the solid protein structure without necessarily filling up the whole pore, indicating high wettability (Supporting Information Videos S4 and S5). Comparison of the dry structure and the partly water-filled structure at the same sample height confirms that water absorption causes distortion of the solid structure (Figure 3g, right, top and bottom). Characteristic structures are displaced to a higher position and away from the radial center (Figure 3g left bottom). The distortion may either result only from slight swelling of the solid matrix or additionally from softening and capillary pressure-induced yielding and stretching of the solid structure.

The complex wetting, capillary absorption and diffusion mechanisms into the pores and the solid matrix with both water and non-watery liquid could be further investigated by fast time-resolved tomographic imaging of the liquid uptake dynamics during absorption and swelling in combination with Washburn experiments.

The presented mixed and high wettability does not only allow to utilize the protein sponge for applications related to absorption of either polar or non-polar liquids but also for subsequent absorption or simultaneous absorption and release of different types of liquids. This shows to be of interest for wound care, coupling drainage absorption with drug release in wound care, or as micronutrient delivery system in functional food products. Besides being abundant and inexpensive, whey protein isolate was demonstrated to allow fibroblast and keratinocyte attachment and growth [43]. Hence, the presented dual-scale porous and liquid-absorbing edible structure with open and elongated macro-pores has an application prospect as scaffold in tissue engineering for biomedical as well as food applications. The production process allows to tune pore size, porosity and mechanical properties by adapting both formulation and process parameters in the foaming and the drying process, such as foam bubble size, gas volume fraction or microwave power. Thereby, the dry protein sponge can be tailored and optimized to the respective field of application (WO/2021/037325, WO/2021/038047). 

## 4. Conclusions

We have demonstrated a simple fabrication of a novel protein-based edible sponge with mixed and high wettability. The fabrication process is based on tailored gas fraction foaming of highly concentrated protein dispersions, followed by controlled microwave-convection drying. This process is cleaner and more efficient than existing alternatives as it does neither require additives, nor solvent exchange or several phase transitions such as in freeze-drying. Moreover, it is scalable and allows continuous production, as the combined unit operations are already utilized in food and related industries. The protein sponge consists of an irreversibly cross-linked structure composed of large open pores surrounded by interconnected micro-porous solid lamellae with high mechanical stability and with the ability to readily absorb and release water, oils and other solvents. Results showed that the water absorption capacity is higher than oil, due to swelling when the water penetrates the porous structure. The water acts as plasticizer, making the material soft and plastically deformable. Whilst absorption of oil preserves the mechanical stability of the porous material. Observation of the dynamics of the liquid absorption using µ-CT indicates that the non-polar liquids are rapidly spread as a film on the solid protein structure, instead polar-liquids may penetrate the solid matrix on a molecular level. The mixed wettability, the open-porous structure and the non-toxic, biodegradable, edible nature of proteins like whey protein isolate allow to target various pharmaceutical, biomedical, and food applications involving absorption and release or scaffolding.

## 5. Patents

Windhab, E. J.; Malafronte, L.; Foschini, S.; Wemmer, J., (ETH Zurich), WO/2021/037325, Method of making a porous sponge-like formulation, a porous sponge-like formulation, use of porous sponge-like formulation and a product comprising the foamed sponge-like formulation, 2019.

Leser, M.; Michel, M.; Windhab, E. J.; Foschini, S.; Wemmer, J.; Malafronte, L.; (Nestlé S.A.), WO/2021/038047, Juicy Sponge Food Product, 2019.

## Data Availability

The data presented in this study are available on request from the first author (J.W.). The data are not publicly available because stored on personal servers.

## Supplementary Materials

The following are available online at https://www.mdpi.com/article/10.3390/ma14092298/s1, Video S1: Real-time absorption of water stained in blue into the protein sponge structure leading to swelling and softening, Video S2: Real-time absorption of silicon oil into the 100 W/60 °C protein sponge structure, remaining stiff and brittle, Video S3: Real-time absorption of ethanol stained with Nile red into the 100 W/60 °C protein sponge structure, remaining stiff and brittle, Video S4: Rotation of the volume-representation of the binarized μ-CT images showing th e 100 W/60 °C protein sponge structure (grey) wetted by decane (red), Video S5: Build-up of the volume-representation of the binarized μ-CT images from bottom to top with the 100 W/60 °C protein sponge structure in grey and decane in red showing that some pores are completely filled with decane while others are only wetted along the solid surfaces, Figure A: Macro- and microstructure of the protein sponge dried at 100 W/60 °C: (top) surface layer; (bottom) inner structure.
