# Supplementary figures and images for: Fabrication of a Novel Protein Sponge with Dual-Scale Porosity and Mixed Wettability Using a Clean and Versatile Microwave-Based Process
Source: Materials (Basel). 2021 Apr 29;14(9):2298. doi: 10.3390/ma14092298 (PMC8124266; doi:10.3390/ma14092298)

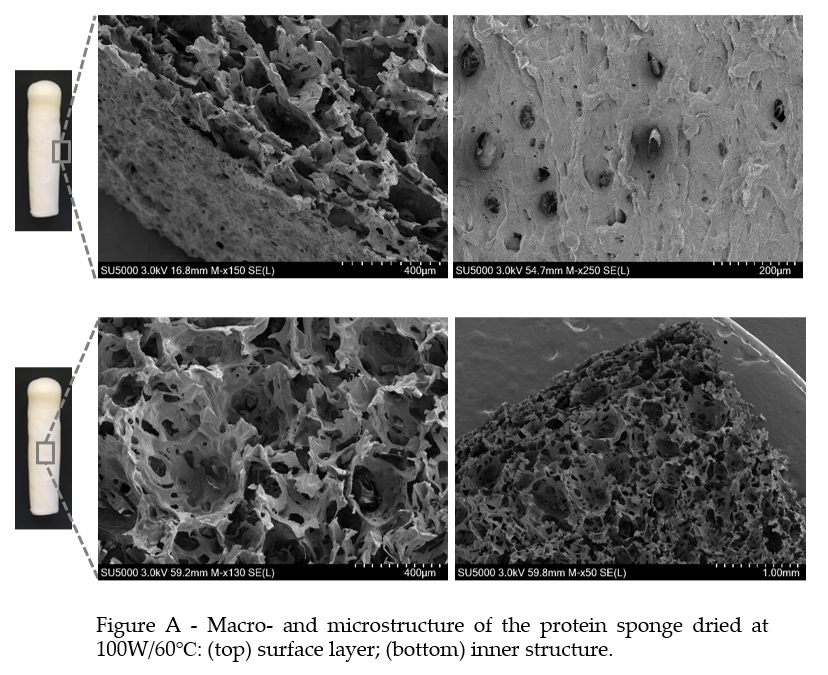

Supplement: Supplementary file 1 [file materials-14-02298-s001.zip › Figure A_revised.tif]
